# The association between dietary acid load and adiposity measures among children and adolescents

**DOI:** 10.1186/s12887-022-03541-6

**Published:** 2022-08-13

**Authors:** Nasim Sorraya, Arman Arab, Shokoofeh Talebi

**Affiliations:** 1 Department of Community Nutrition, School of Nutrition and Food Science, Food Security Research Center, Isfahan University of Medical Sciences, Isfahan, Iran; 2 Department of Clinical Nutrition, School of Nutrition and Food Science, Food Security Research Center, Isfahan University of Medical Sciences, Hezar Jerib street, Isfahan, 8174673461 Iran

**Keywords:** Dietary acid load, Obesity, Diet, Anthropometric indices

## Abstract

**Background:**

Childhood obesity is one of the most important health problems in the present century. It is imperative to plan preventive programs through risk factor identification. The present study was conducted to examine the association between the dietary acid load (DAL) and anthropometric indices in a sample of Iranian children and adolescents.

**Methods:**

In the current survey, students aged 11–18 years were recruited. To assess usual dietary intake, a validated self-administered 168-item food frequency questionnaire was used. Potential renal acid load (PRAL) and net endogenous acid production (NEAP) was used to estimate DAL. The association between DAL and anthropometric indices was evaluated using logistic regression and reported as an odds ratio (OR) with a 95% confidence interval (CI).

**Results:**

In total, 456 students (267 girls), with a mean age of 14.44 years participated in the current study. After controlling for potential confounders, the PRAL was positively associated with the risk of obesity (OR = 4.56, 95% CI: 2.26, 9.17), abdominal obesity (OR = 12.64, 95% CI: 3.05, 52.27), and adiposity based on the percent of body fat (PBF) (OR = 3.58, 95% CI: 1.83, 6.99). The NEAP was also significantly associated with the risk of obesity (OR = 5.17, 95% CI: 2.56, 10.43), abdominal obesity (OR = 15.08, 95% CI: 3.35, 67.81), and adiposity based on PBF (OR = 4.53, 95% CI: 2.30, 8.92).

**Conclusion:**

Our findings suggest that higher adherence to the acidogenic diet, expressed as DAL, was associated with a higher risk of general and central obesity among children and adolescents.

## Introduction

World Health Organization (WHO) identifies obesity among children and adolescents as one of the most important health issues in the present century [1]. Both developed and developing countries are struggling with this crisis [2, 3]. Iran is among developing countries facing an increase in the prevalence of obesity [4], with 5.82% of the Iranian students suffering from this problem, according to a meta-analysis in 2017 [5]. It is imperative to plan preventive programs through risk factor identification since the increased prevalence of pediatric obesity consequently leads to increased rates of diabetes, metabolic syndrome, cardiovascular events, and other chronic obesity-related disorders [3].

Among preventive strategies, modifying dietary-related behaviors is considered to be one of the most effective ones [6]. In recent years, the adoption of a western diet (high amounts of animal foods and a lower intake of fruits and vegetables) among children and adolescents has been documented in Iran [7]. This can lead to a condition called diet-induced acidosis [8]. Acidosis or increased acid loading due to the consumption of animal foods has been associated with various health conditions including chronic kidney diseases, hypertension (HTN), and impaired bone metabolism [9]. More recently, the potential influence of dietary acid load (DAL) on adiposity indices has been suggested [10].

DAL is composed of two scores including the potential renal acid load (PRAL) and the net endogenous acid production (NEAP). These scores are based on protein, phosphorous, calcium, magnesium, and potassium intake validated according to the estimated equivalents in the 24-h urine measurement [10]. Numerous studies are available in adults reporting the association between anthropometric indices and DAL [11–14]. However, according to our literature search, studies on children especially in middle-eastern countries are scarce. For example, a large cross-sectional study showed that NEAP was positively associated with neck circumference in students aged 6–18 years. Also, higher PRAL and NEAP were associated with lower body mass index (BMI) in parents [15]. In addition, findings from another study revealed that there was no association between PRAL and anthropometric measures in 6-year-old girls [16].

Due to limited evidence on the association between DAL and adiposity measures in children and adolescents living in developing countries, the present study aimed at examining the association between DAL, based on PRAL and NEAP, and adiposity-related indices among children and adolescents in Iran.

## Methods

### Ethical standards

The ethics committee of Isfahan University of Medical Sciences approved the study protocol (IR. MUI.REC.1394.3/294) which was conducted according to the principles of the Declaration of Helsinki. The objectives and protocol of the study were explained to the students and their parents. Those who verbally agreed to participate signed informed written consent.

### Study design and population

In the current cross-sectional survey, students aged 11–18 years were recruited. From September 2015 to February 2016, eligible subjects were recruited from 13 schools in four different districts of Isfahan, Iran, using a random cluster sampling method. Subjects on any medication or special diet or suffering from any type of disease were excluded. A total of 456 out of 500 invited individuals completed the study (response rate of 91.2%).

### Anthropometric assessment

To calculate body mass index (BMI), students were weighed on a digital scale (Omron Corp, Kyoto, Japan) and a non-stretchable tape was used for height estimation. Both measurements were performed without any excessive clothing and shoes and recorded to the nearest 100 g and 0.5 cm, respectively. Based on the international obesity taskforce BMI reference cut-offs [17], two categories were defined: underweight/ normal weight subjects or overweight/obese children. Those with a waist-to-height ratio (WHtR) of ≥ 0.5 were defined as having abdominal obesity [18]. Authors also recorded the percent body fat (PBF) shown on the Omron BF511 body composition monitor (Omron Corp, Kyoto, Japan) to define adiposity risk as follows: PBF > 25% for boys and > 35% for girls aged 11 years and older [19].

### Dietary assessment

To assess usual dietary intake, a validated self-administered semi-quantitative 168-item Willett-format food frequency questionnaire (FFQ) was completed through a face-to-face interview by an expert dietitian. This questionnaire includes a list of food items and a standard serving size for each [20]. Students were asked to report how frequently they consumed a given serving of each food item during the past year on the basis of daily, weekly, or monthly. To calculate the grams of portion sizes of consumed foods, household measures were used [21]. Nutritionist IV software (First Databank, Hearst Corp, San Bruno, CA, USA) was utilized for dietary analysis.

### DAL calculations

DAL was approximated using PRAL and NEAP based on the following formulas, respectively [15]:

$$\text{PRAL (mEq/d)}=0.4888\times\text{protein intake (g/d)}+0.0366\times\text{phosphorus (mg/d)}-0.0205\times\text{potassium (mg/d)}-0.0125\times\text{calcium (mg/d)}-0.0263\times\text{magnesium (mg/d)}$$

$$\text{NEAP (mEq/d)}=\lbrack54.5\times\text{protein intake (}\textit{g}\text{/}\textit{d}\text{)}\div\text{potassium intake (mEq/d)}\rbrack-10.2.$$

### Assessment of other variables

The authors collected data about socio-demographic parameters such as sex, age, income, parental education level, and past medical history using a general questionnaire. Overall physical activity level during the past 7 days was estimated by the Physical Activity Questionnaire (PAQ) with invariably high validity and moderate reliability [22]. The mean score of nine items, each scored on a 5-point scale, presents a summary physical activity score which is interpreted as follows: 1 = low physical activity, 2–4 = moderate physical activity, and 5 = high physical activity.

### Statistical analysis

All of the statistical analyses were carried out using the SPSS software version 25 (IBM Corp, Armonk, NY, USA). A *P*-value < 0.05 considered statistically significant. The normality of continuous variables was assessed using Kolmogorov–Smirnov and Q-Q plot. The continuous and categorical variables were shown as mean ± standard deviation (SD) and number (percent), respectively. The difference of continuous variables across tertiles of the PRAL and NEAP was assessed using the one-way analysis of variance (ANOVA). The distribution of categorical variables through the tertiles of the PRAL and NEAP was examined using the Chi-square test. To investigate the association between the PRAL and NEAP with adiposity measures, multinomial binary logistic regression analysis was implemented in different models and an odds ratio (OR) with a 95% confidence interval (CI) was reported. First, we adjusted for age (continuous) and sex. In the second model, further adjustment was made for household income (very low, low, moderate, high), parent’s education (illiterate, diploma or lower, university degree), sleep hours (continuous), and physical activity (low, moderate, high). In the last model, total energy intake was controlled to obtain an energy-independent association between DAL and obesity.

## Results

Overall, 456 children and adolescents aged 11–18 years make up our study population with a mean (SD) age of 14.44 (2.09) and a mean BMI of 20.88 (4.22). The mean (SD) NEAP and PRAL of the study population were 41.72 (9.62) and -1.79 (9.04), respectively. The general characteristics and anthropometric measures of the study population across tertiles of PRAL and NEAP are presented in Table 1. Participants in the highest tertile of PRAL, compared to the lowest tertile, had higher weight, BMI, WHtR, PBF, abdominal obesity, adiposity by PBF, PRAL, and NEAP. Furthermore, they were more likely to be older, overweight/obese, and had low physical activity. Those in the top tertile of NEAP were more likely to be overweight/obese and boys with low physical activity, compared to the lowest tertile. In addition, they had higher weight, height, BMI, WHtR, PBF, abdominal obesity, adiposity by PBF, PRAL, and NEAP.

**Table 1:** General characteristics and anthropometric measures of study population across tertiles of PRAL and NEAP

	**Tertiles of PRAL**				**Tertiles of NEAP**			
	< -4.30 mEq/d	-4.30 to 2.22 mEq/d	> 2.22 mEq/d	***P* value**	< 37.40 mEq/d	37.40 to 44.86 mEq/d	> 44.86 mEq/d	***P* value**
N	152	152	152		152	152	152	
Age (y)	14.44 ± 2.16	14.11 ± 2.08	14.78 ± 1.98	0.018	14.33 ± 2.14	14.25 ± 2.03	14.75 ± 2.08	0.082
Sex				0.295				0.027
Girls	98 (64.5)	80 (52.6)	89 (58.6)		103 (67.8)	80 (52.6)	84 (55.3)	
Boys	54 (35.5)	72 (47.4)	63 (41.4)		49 (32.2)	72 (47.4)	68 (44.7)	
Physical activity				0.002				0.001
Low	45 (29.6)	47 (30.9)	71 (46.7)		44 (28.9)	47 (30.9)	72 (47.4)	
Moderate	107 (70.4)	105 (69.1)	81 (53.3)		108 (71.1)	105 (69.1)	80 (52.6)	
High	0 (0.0)	0 (0.0)	0 (0.0)		0 (0.0)	0 (0.0)	0 (0.0)	
Weight (kg)	50.86 ± 11.95	50.83 ± 13.09	57.86 ± 16.88	< 0.001	49.70 ± 11.49	51.25 ± 12.82	58.60 ± 17.03	< 0.001
Height (cm)	158.14 ± 10.50	157.77 ± 11.28	160.11 ± 10.45	0.125	157.10 ± 9.98	158.46 ± 11.48	160.46 ± 10.62	0.024
BMI (kg/m^2^)	20.13 ± 3.51	20.19 ± 3.75	22.33 ± 4.91	< 0.001	19.95 ± 3.53	20.19 ± 3.57	22.52 ± 4.94	< 0.001
WHR	0.81 ± 0.05	0.81 ± 0.05	0.82 ± 0.06	0.056	0.81 ± 0.05	0.81 ± 0.05	0.83 ± 0.06	0.006
PBF (%)	23.82 ± 10.01	23.38 ± 9.43	26.91 ± 10.26	0.005	23.86 ± 10.06	23.13 ± 9.19	27.10 ± 10.34	0.001
BMI category				< 0.001				< 0.001
Normal	127 (83.6)	115 (75.7)	95 (62.5)		127 (83.6)	120 (78.9)	90 (59.2)	
Overweight/obese	25 (16.4)	37 (24.3)	57 (37.5)		25 (16.4)	32 (21.1)	62 (40.8)	
Abdominal obesity				< 0.001				< 0.001
Normal	147 (96.7)	143 (94.1)	126 (82.9)		148 (97.4)	144 (94.7)	124 (81.6)	
Obese	5 (3.3)	9 (5.9)	26 (17.1)		4 (2.6)	8 (5.3)	28 (18.4)	
Adiposity by PBF				< 0.001				< 0.001
Normal	127 (83.5)	115 (75.6)	92 (60.5)		129 (84.9)	119 (78.3)	87 (57.2)	
Obese	25 (16.5)	37 (24.4)	60 (39.5)		23 (15.1)	33 (21.7)	65 (42.8)	
PRAL (mEq/d)	-11.65 ± 7.12	-0.78 ± 1.88	7.06 ± 3.78	< 0.001	-11.26 ± 7.51	-0.90 ± 2.87	6.80 ± 4.04	< 0.001
NEAP (mEq/d)	31.96 ± 4.70	41.28 ± 3.00	51.93 ± 6.88	< 0.001	31.72 ± 4.39	41.12 ± 2.07	52.33 ± 6.41	< 0.001

Data are presented as mean ± standard deviation or number (percent)

*P*-value obtained from chi-square analysis for categorical variables and analysis of variance (ANOVA) for continuous variables

*BMI* Body Mass Index, *WHtR* Waist to Hip Ratio, *PBF* Percent of Body Fat, *PRAL* Potential Renal Acid Load, *NEAP* Net Endogenous Acid Production

Individuals in the top tertile of PRAL, compared to the lowest tertile, significantly consumed higher amounts of protein and lower amounts of magnesium and potassium. They also tended to consume higher total energy and carbohydrate. Children and adolescents with the highest tertile of NEAP had a significantly higher dietary intake of protein and lower intake of potassium, calcium, and magnesium (Table 2).

**Table 2:** Dietary intakes of study population across tertiles of PRAL and NEAP

	**Tertiles of PRAL**				**Tertiles of NEAP**			
	< -4.30 mEq/d	-4.30 to 2.22 mEq/d	> 2.22 mEq/d	***P* value**	< 37.40 mEq/d	37.40 to 44.86 mEq/d	> 44.86 mEq/d	***P* value**
***Macronutrients***								
Total energy (kcal/d)	1690.94 ± 308.60	1642.56 ± 308.65	1720.97 ± 302.01	0.081	1654.82 ± 293.92	1677.98 ± 321.55	1721.67 ± 304.57	0.157
Carbohydrate (g/d)	256.87 ± 48.50	246.70 ± 48.49	259.07 ± 51.99	0.069	250.64 ± 46.52	251.69 ± 50.35	260.31 ± 52.32	0.180
Protein (g/d)	56.59 ± 11.72	57.31 ± 11.28	60.78 ± 12.09	0.004	54.72 ± 10.94	59.22 ± 11.70	60.74 ± 12.03	< 0.001
Fat (g/d)	53.63 ± 14.11	50.95 ± 14.02	51.54 ± 10.76	0.174	53.13 ± 13.75	51.86 ± 14.69	51.13 ± 10.42	0.401
***Micronutrients***								
Potassium (mg/d)	2878.51 ± 636.09	2369.55 ± 468.31	2100.09 ± 457.95	< 0.001	2808.84 ± 654.51	2456.64 ± 503.14	2082.67 ± 448.59	< 0.001
Magnesium (mg/d)	223.04 ± 44.91	199.30 ± 40.69	186.77 ± 40.80	< 0.001	218.86 ± 44.57	206.50 ± 41.95	183.76 ± 40.45	< 0.001
Calcium (mg/d)	896.87 ± 227.82	869.07 ± 227.84	853.87 ± 243.22	0.265	879.52 ± 233.39	904.19 ± 222.51	836.10 ± 239.89	0.036
Phosphorous (mg/d)	1004.60 ± 218.61	980.34 ± 219.64	983.37 ± 229.71	0.586	992.22 ± 215.37	1017.38 ± 222.04	958.70 ± 227.42	0.070

Data are presented as mean ± standard deviation

*P* < 0.05 was considered statistically significant

*P*-value obtained from analysis of variance (ANOVA)

The odds ratio and 95% CI for adiposity measures of the study population across categories of PRAL and NEAP are provided in Table 3. In the crude model, PRAL was positively associated with the risk of obesity (OR = 3.04, 95% CI: 1.77, 5.23) for those in the highest tertile of PRAL compared to the lowest tertile. This association remained also significant after further adjustment for sex, age, household income, parent’s education, sleep hours, physical activity, and total energy intake (OR = 4.56, 95% CI: 2.26, 9.17). After adjustment for potential confounders, the multivariable odds ratio for the risk of abdominal obesity was 1.00 (reference), 2.82 (95% CI: 0.60, 13.20), and 12.64 (95% CI: 3.05, 52.27) across increasing tertiles of PRAL. The risk of adiposity assessed by PBF was significantly associated with PRAL even after adjustment for potential confounders (OR = 3.58, 95% CI: 1.83, 6.99) for those in the top tertile of PRAL compared to the lowest tertile.

**Table 3:** Odds ratio and 95% confidence interval for adiposity measures across tertiles of PRAL and NEAP

	**Tertiles of PRAL**				**Tertiles of NEAP**			
	< -4.30 mEq/d	-4.30 to 2.22 mEq/d	> 2.22 mEq/d	**P trend**	< 37.40 mEq/d	37.40 to 44.86 mEq/d	> 44.86 mEq/d	**P trend**
**Overweight/obesity**								
Crude	Ref	1.63 (0.92, 2.88)	3.04 (1.77, 5.23)	< 0.001	Ref	1.35 (0.75, 2.41)	3.50 (2.04, 5.98)	< 0.001
Model 1	Ref	1.58 (0.89, 2.81)	3.28 (1.89, 5.70)	< 0.001	Ref	1.37 (0.76, 2.47)	3.91 (2.24, 6.80)	< 0.001
Model 2	Ref	1.50 (0.82, 2.77)	2.89 (1.62, 5.15)	< 0.001	Ref	1.29 (0.69, 2.39)	3.39 (1.89, 6.07)	< 0.001
Model 3	Ref	2.30 (1.11, 4.76)	4.56 (2.26, 9.17)	< 0.001	Ref	1.48 (0.71, 3.09)	5.17 (2.56, 10.43)	< 0.001
**Abdominal obesity**								
Crude	Ref	2.31 (0.69, 7.68)	7.58 (2.57, 22.31)	< 0.001	Ref	2.74 (0.71, 10.53)	11.14 (3.30, 37.52)	< 0.001
Model 1	Ref	2.17 (0.65, 7.25)	7.85 (2.65, 23.26)	< 0.001	Ref	2.67 (0.69, 10.35)	11.65 (3.42, 39.61)	< 0.001
Model 2	Ref	1.71 (0.46, 6.34)	6.99 (2.15, 22.72)	< 0.001	Ref	2.59 (0.62, 10.76)	10.32 (2.78, 38.22)	< 0.001
Model 3	Ref	2.82 (0.60, 13.20)	12.64 (3.05, 52.27)	< 0.001	Ref	2.42 (0.48, 12.21)	15.08 (3.35, 67.81)	< 0.001
**Adiposity by PBF**								
Crude	Ref	1.68 (0.93, 3.01)	3.42 (1.96, 5.98)	< 0.001	Ref	1.61 (0.87, 2.96)	4.48 (2.53, 7.90)	< 0.001
Model 1	Ref	1.68 (0.93, 1.03)	3.33 (1.90, 5.83)	< 0.001	Ref	1.60 (0.86, 2.95)	4.35 (2.45, 7.72)	< 0.001
Model 2	Ref	1.72 (0.91, 3.24)	2.96 (1.62, 5.40)	< 0.001	Ref	1.62 (0.85, 3.10)	3.90 (2.11, 7.20)	< 0.001
Model 3	Ref	2.27 (1.13, 4.57)	3.58 (1.83, 6.99)	< 0.001	Ref	1.70 (0.83, 3.46)	4.53 (2.30, 8.92)	< 0.001

Data are presented as odds ratio (95% confidence interval)

^†^*P* < 0.05 was considered statistically significant

Crude: Unadjusted

Model 1: adjusted for age and sex

Model 2: Model 1 + household income, parent’s education, sleep hours, and physical activity

Model 3: Model 2 + adjusted for total energy intake

The NEAP was significantly associated with the risk of overweight/obesity for those in the greatest tertile of NEAP compared to the lowest tertile before (OR = 3.50, 95% CI: 2.04, 5.98) and after controlling for potential confounders (OR = 5.17, 95% CI: 2.56, 10.43). The risk of abdominal obesity was significantly associated with NEAP for participants in the highest tertile of NEAP compared to the lowest tertile (OR = 11.14, 95% CI: 3.30, 37.52). Further adjustment for sex, age, household income, parent’s education, sleep hours, physical activity, and total energy intake did not change the overall finding for NEAP and the risk of abdominal obesity (OR = 15.08, 95% CI: 3.35, 67.81). After controlling for confounders, the multivariable odds ratio for the risk of adiposity assessed by PBF were 1.00 (reference), 1.70 (95% CI: 0.83, 3.46), and 4.53 (95% CI: 2.30, 8.92) across increasing tertiles of NEAP.

## Discussion

We found that the DAL, assessed by the PRAL and NEAP, was significantly associated with a greater risk of overweight/obesity, abdominal obesity, and adiposity by PBF. There are previous studies on the relationship between the DAL and risk of obesity; however, this issue has been examined scarcely among Iranian children and adolescents. A population-based cross-sectional study among 5326 Iranian children and adolescents (6–18 years) demonstrated a direct association between the neck circumference, an index of upper subcutaneous adipose tissue distribution, and NEAP. However, this study failed to show any association between the NEAP and PRAL with other anthropometric measures [15]. Similarly, another cross-sectional study among 1136 young Japanese women (18–22 years) revealed a positive association between DAL, estimated as the ratio of dietary protein to K (Pro: K), with BMI and waist circumference [23]. In line with our findings, another cohort study among the aged Swedish individuals also proposed a positive association between the PRAL and BMI in both women and men [24]. Similarly, another investigation among the 547 patients diagnosed with diabetic nephropathy also suggested the association between DAL, assessed by Pro: K, and waist circumference [25]. Likewise, Mozaffari and colleagues reported a positive relationship between DAL and waist circumference but not BMI, among 371 Iranian women (20–50 years). In line with Mozaffari et al.’s findings, another observational study among Japanese employees also demonstrated no association between DAL and BMI [26]. The inconsistencies in the available literature can be attributed to the observed differences in race and ethnicity, sample size, age-range and health status of the study population, dietary habits, and lifestyle of participants.

Despite the previous documents in the field of DAL and adiposity measures, the exact mechanism is not fully understood. A previous investigation by Dawson-Hughes et al. suggested that acidogenic diets may lead to loss of lean body mass through increased amino-acid oxidation, proteolysis, and decreased protein synthesis via an alteration in IGF signaling or ubiquitin–proteasome system [27]. Moreover, the adipogenic effects of the higher dietary acid load have been suggested previously [28].

Most macro-/micronutrients are associated with total energy intake [29]. This fact is because the individuals with higher total energy intake, on average, also eat more of all macro-/micronutrients. Moreover, most nutrients are contributed directly to total energy intake. Therefore, we controlled total energy intake as a surrogate marker of dietary intake to provide an independent association [29].

The epidemiology of nutrition has been criticized over the years for its reliance on observation and inaccurate dietary assessment [30]. Although, there is no perfect method to assess all aspects of human diet and eating behavior, concerns regarding dietary measurement have been resolved using standardized questionnaires and strict methods [31]. We implemented a rigorous methodology in terms of patient selection, sampling method, and the use of a valid and reliable questionnaire [32] to improve the internal validity of the current study. In terms of external validity, these findings can be generalized to the overweight/obese Iranian children and adolescents; however, results must be generalized with caution to the other population.

Although the present cross-sectional study is among the few reports on the association between DAL and adiposity measures among Iranian children and adolescents, some limitations should be noted. Our findings propose that the association between the PRAL and NEAP with anthropometric indices is independent of potential confounders including sex, age, household income, parent’s education, sleep hours, physical activity, and total energy intake; however, there are also residual confounders which their effects cannot be excluded. Therefore, further research on controlling additional confounders is suggested. Due to the cross-sectional design of the current study, the causality of the recent relationship remained unclear. Moreover, also we used a validated version of a 168-item FFQ, the likelihood of misclassification might influence our findings. Besides, the PRAL and NEAP were estimated from dietary intake, instead of being measured directly. However, FFQ-derived PRAL and NEAP were shown to be correlated with 24-h urinary values and also widely implemented in previous epidemiological studies [33]. In addition, no data was available in terms of the renal function of participants which is an imperative organ in the regulation of acid–base balance. However, since the participants were healthy children, it can be assumed that they had normal renal function. Moreover, the DAL was estimated using the FFQ which is a semi-quantitative approach, rather than calculation on the basis of meal analysis or food diary.

## Conclusion

In conclusion, our findings suggest that higher adherence to the acidogenic diet, expressed as DAL, was associated with a higher risk of general and central obesity among children and adolescents. Further prospective cohort studies are called to investigate a cause-and-effect association between DAL and adiposity measures and also to clarify the underlying mechanisms. Moreover, further interventional studies are needed to implement an alkaline diet in overweight/obese individuals.

## Data Availability

The data that support the findings of this study are available from the corresponding author upon reasonable request.
